# Accuracy and feasibility in building a personalized 3D printed femoral pseudoaneurysm model for endovascular training

**DOI:** 10.1371/journal.pone.0304506

**Published:** 2024-06-03

**Authors:** Suat Yee Lee, Shen Cheak Currina Chew, Pei Hua Lee, Hung Da Chen, Shao Min Huang, Chun Hung Liu, Fatt Yang Chew

**Affiliations:** 1 Department of Pathology, Chung Shan Medical University Hospital, Taichung, Taiwan; 2 Department of Pathology, School of Medicine, Chung Shan Medical University, Taichung, Taiwan; 3 Faculty of Social Sciences & Humanities, University of Technology, Johor Bahru, Malaysia; 4 Department of Medical Imaging, China Medical University Hospital, Taichung, Taiwan; 5 Department of Medicine, Show Chwan Memorial Hospital, Chang Hua, Taiwan; 6 Department of Radiology, School of Medicine, China Medical University, Taichung, Taiwan; Public Library of Science, UNITED KINGDOM

## Abstract

**Background:**

The use of three-dimensional(3D) printing is broadly across many medical specialties. It is an innovative, and rapidly growing technology to produce custom anatomical models and medical conditions models for medical teaching, surgical planning, and patient education. This study aimed to evaluate the accuracy and feasibility of 3D printing in creating a superficial femoral artery pseudoaneurysm model based on CT scans for endovascular training.

**Methods:**

A case of a left superficial femoral artery pseudoaneurysm was selected, and the 3D model was created using DICOM files imported into Materialise Mimics 22.0 and Materialise 3-Matic software, then printed using vat polymerization technology. Two 3D-printed models were created, and a series of comparisons were conducted between the 3D segmented images from CT scans and these two 3D-printed models. Ten comparisons involving internal diameters and angles of the specific anatomical location were measured.

**Results:**

The study found that the absolute mean difference in diameter between the 3D segmented images and the 3D printed models was 0.179±0.145 mm and 0.216±0.143mm, respectively, with no significant difference between the two sets of models. Additionally, the absolute mean difference in angle was 0.99±0.65° and 1.00±0.91°, respectively, and the absolute mean difference in angle between the two sets of data was not significant. Bland-Altman analysis confirmed a high correlation in dimension measurements between the 3D-printed models and segmented images. Furthermore, the accuracy of a 3D-printed femoral pseudoaneurysm model was further tested through the simulation of a superficial femoral artery pseudoaneurysm coiling procedure using the Philips Azurion7 in the angiography room.

**Conclusions:**

3D printing is a reliable technique for producing a high accuracy 3D anatomical model that closely resemble a patient’s anatomy based on CT images. Additionally, 3D printing is a feasible and viable option for use in endovascular training and medical education. In general, 3D printing is an encouraging technology with diverse possibilities in medicine, including surgical planning, medical education, and medical device advancement.

## Introduction

Pseudoaneurysm is a type of false aneurysm that occurs due to localized arterial wall injury and incomplete hemostasis at the injury site. It is an abnormal dilatation or outpouching of a blood vessel caused by partial or complete rupture of the vessel wall. It is characterized by the extravasation of blood outside the arterial wall, which is contained and controlled by a pseudocapsule that forms around the site of injury [1]. Pseudoaneurysm can occur in various types of blood vessels, including arteries and veins, and can be caused by trauma, infection, iatrogenic, or other underlying conditions that damage the blood vessel wall. The femoral artery has been widely utilized and remained as the primary percutaneous arterial access site for non-cardiac percutaneous based procedures. The reported incidence of femoral artery pseudoaneurysms varies significantly in the literature [2–12]. Some guidelines by certain society such as Society of Cardiovascular and Interventional Radiology suggest an acceptable rate of less than 0.2% [5]. In a study of using color flow duplex sonography to examine the puncture site in 565 consecutive patients who had undergone transfemoral arterial catheterization with angiography, percutaneous transluminal angioplasty, or local lysis, the overall incidence of pseudoaneurysm was found to be 7.7% [6]. However, a prospective study of over 1000 patients showed pseudoaneurysms in 3.8% of patients when routine duplex imaging was performed, indicating a higher incidence [7]. Another study conducted in Japan [8] also demonstrated similar results with an incidence of 2.9% when routine ultrasound imaging was performed. These findings highlight the variability in reported incidence rates and the importance of vigilant monitoring and appropriate management in clinical practice.

The use of three-dimensional (3D) printing is broadly across many medical specialties. It is an innovative, and rapidly growing technology to produce custom anatomical models and medical conditions models for medical teaching [13–16], surgical planning, and patient education. 3D printing in medical education [15] has skyrocketed in recent years. Applications within medical education are emerging due to 3D printing’s ability to produce specialized models, devices, and implants that can potentially create a highly realistic virtual medical conditions models that simulate the medical problems that a patient is facing. With the advancement of 3D printing, it could become an alternative to the traditional hands-on training performed on cadavers, and animals. In the literature reported by Sebastian Mafel [14] and Goudie C [16] in using 3D-printed vasculature as medical tools for anatomy learning and understanding the vascular interventional radiology procedure, majority of the feedback are positive. Application of 3D printing in medicine may provide many benefits, therefore understanding the workflow in creating a 3D-printed customized medical condition model is essential. Here, we reported our experience and workflow in creating a 3D printed model that is designed for endovascular simulation of angiography and superficial femoral artery pseudoaneurysm embolization. Moreover, a series of comparisons of diameter and angle between 3D segmented images from the CT and 3D-printed models are evaluated to determine the accuracy and feasibility of the 3D-printed model.

## Materials and methods

### Process in creating 3D-models

**Fig 1:**
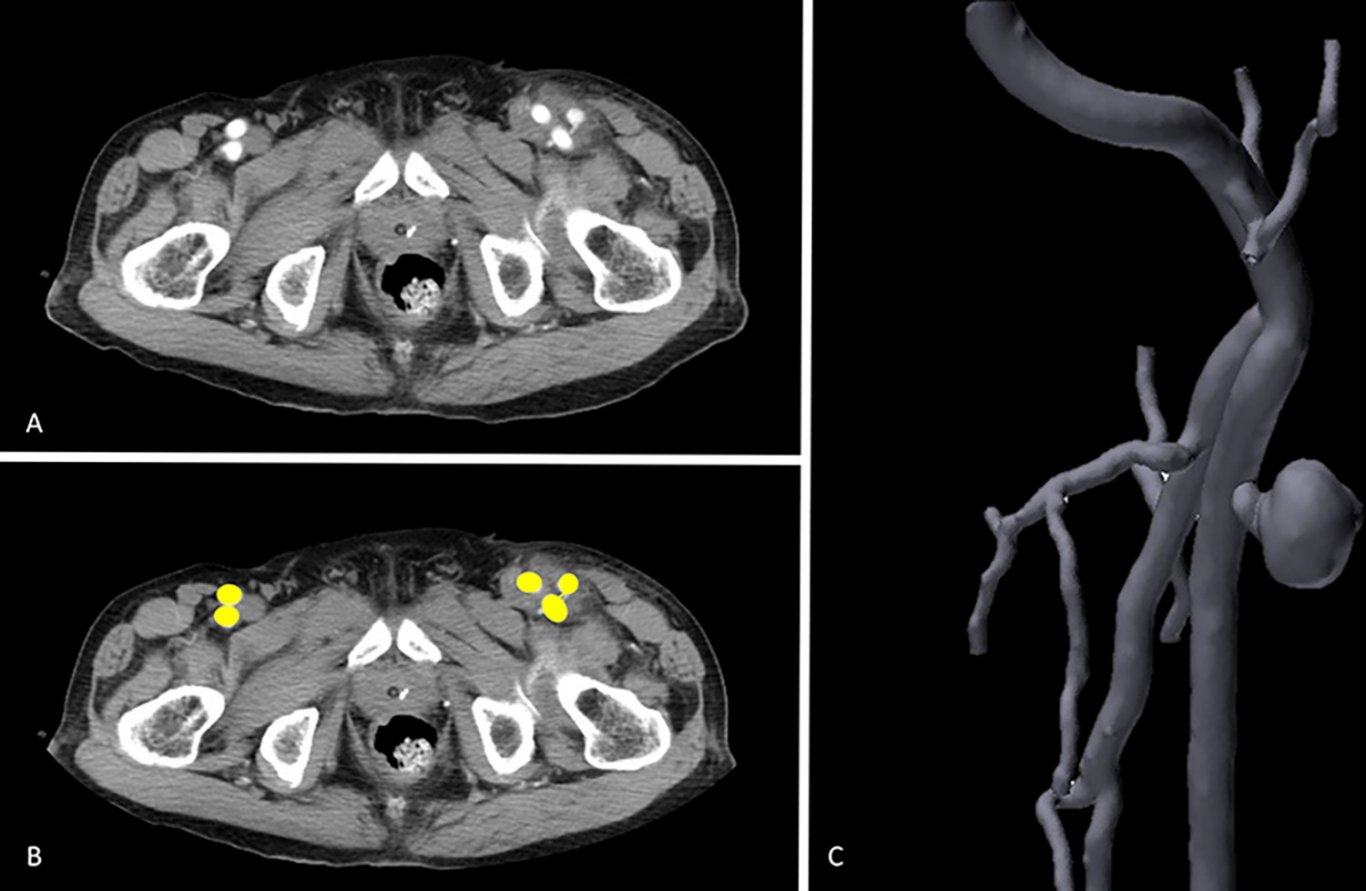
Pelvic Computed Tomography images(CT). The raw data of the pelvic CT with contrast **(A)** stored in the DICOM format was acquired to create the 3D model. **(B)** Then, the DICOM files are imported into Materialise Mimics 22.0 software where relevant arterial anatomy is segmented. (yellow) **(C)**Next the segmented model is exported to Materialise 3-Matic to create mesh representations of the individual parts or volumes to be printed.

This study was approved by our Department of Education, Research Ethical Committee. For the relevant guidelines and regulation in our study is performed in accordance with relevant guidelines and regulations. This study commenced on March 01, 2021, and concluded on November 31, 2021. In this study, a case of a left superficial femoral artery pseudoaneurysm was selected to be printed in this research. First, the raw data of the pelvic computed tomography(CT) with contrast **(Fig 1A)** stored in the Digital Imaging and Communications in Medicine(DICOM) format was acquired to create the 3D model. The CT was performed using a GE CT scanner (Optima CT660 scanner; GE Healthcare, Milwaukee, WI)) under the following imaging protocols: scan type: axial, slice thickness 0.625 mm, gantry rotation time 0.8 s, large FoV, mA: 250–400, 120kVp and standard reconstruction algorithm. During scanning, the non-ionic contrast medium Omnipaque 300 mgI/mL (IOHEOL 32.35 g/100 mL) with injection rate 3ml/s was used. Then, the DICOM files of arterial phases of CT images were imported into Materialise Mimics 22.0 (Materialise, Leuven, Belgium) software where relevant arterial anatomy is segmented(**Fig 1B, yellow**). Next, the segmented model was exported to Materialise 3-Matic (Materialise, Leuven, Belgium), a CAD software for creating mesh representations of the individual volumes of this model to be printed **(Fig 1C)**. After the file was processed, it was uploaded to Phrozen’s 3D printer, and the model was printed using vat polymerization technology layer by layer. The thickness of each layer was set to 100 μm, and the printer took 8 seconds to complete each layer using resin. The printing process took 96 hours to complete. The vascular wall thickness was set to 1.0 mm during printing to ensure durability. The model was positioned straight to prevent the formation of internal supports within the vessels. Once printing was complete, the support structures were removed with a scraper knife and blade. Finally, the model was successfully created. Both models were created using a similar method.

### Analysis of the diameter and angle between 3D segmented images and 3D-printed models

**Fig 2:**
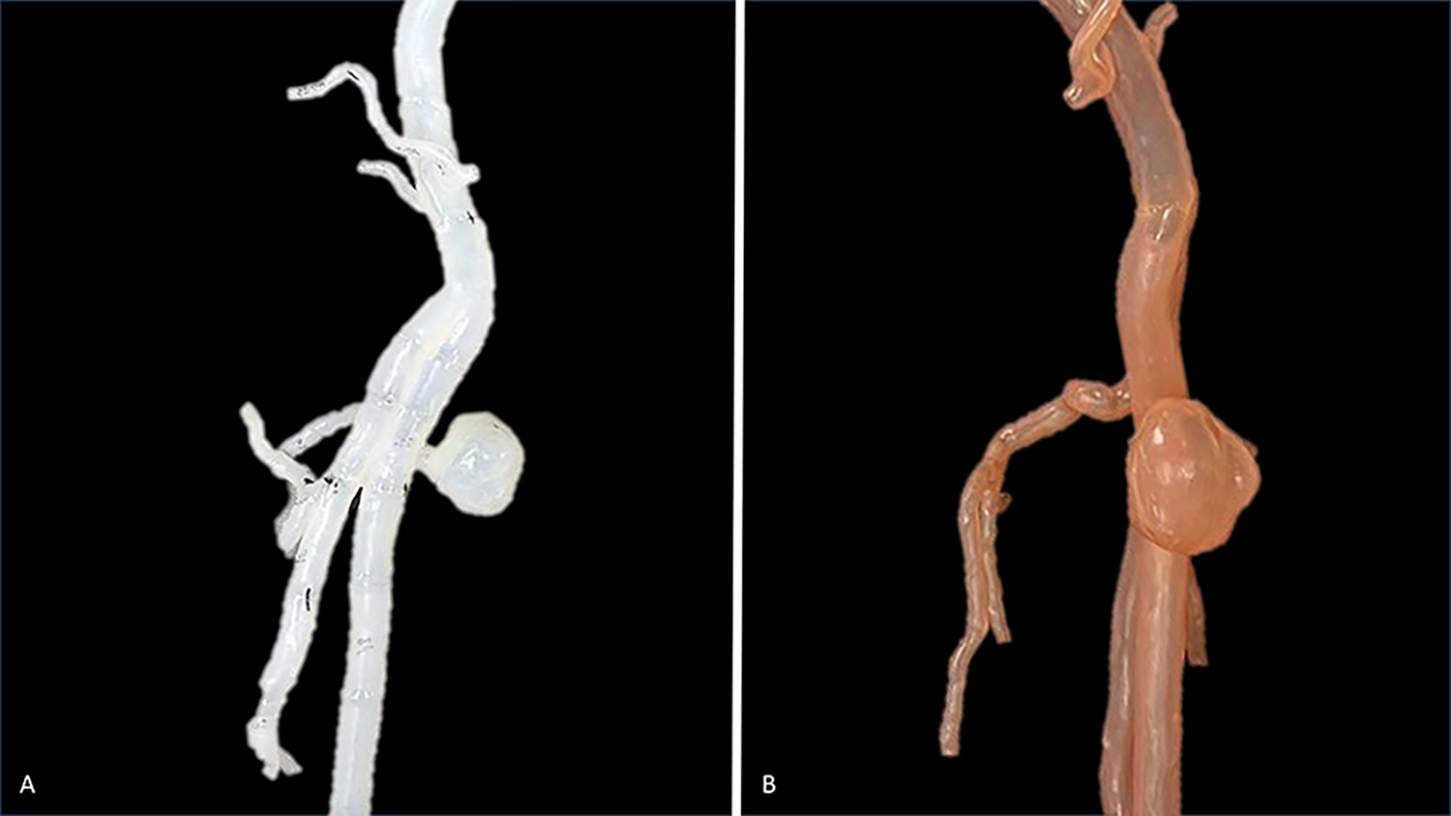
Two superficial femoral artery pseudoaneurysm 3D printed models.

**Fig 3:**
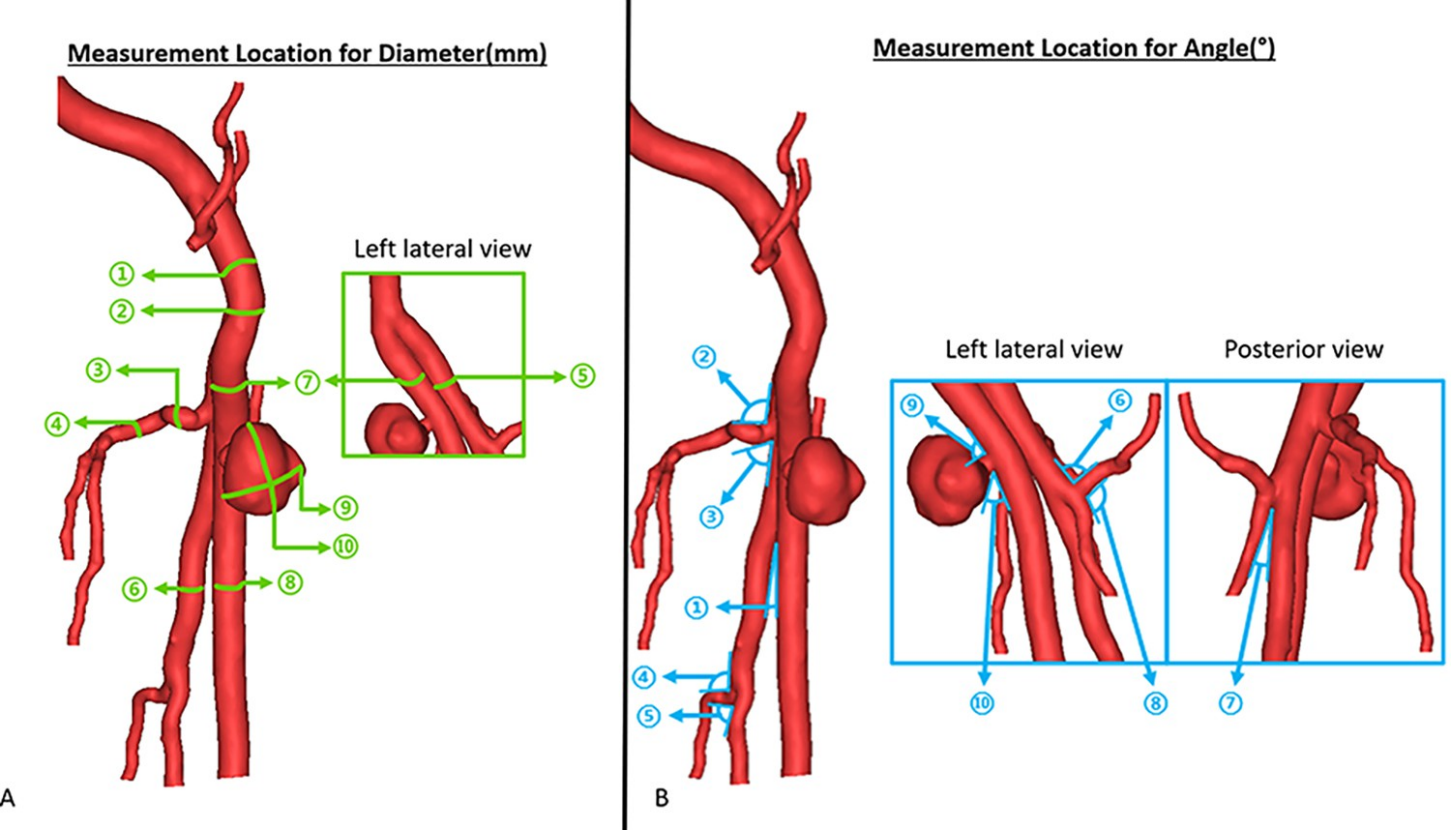
Ten specific anatomical location for measurement of **(A)** diameters and **(B)** angle to assess the accuracy of the segmented models and the two printed 3D models.

**Fig 4:**
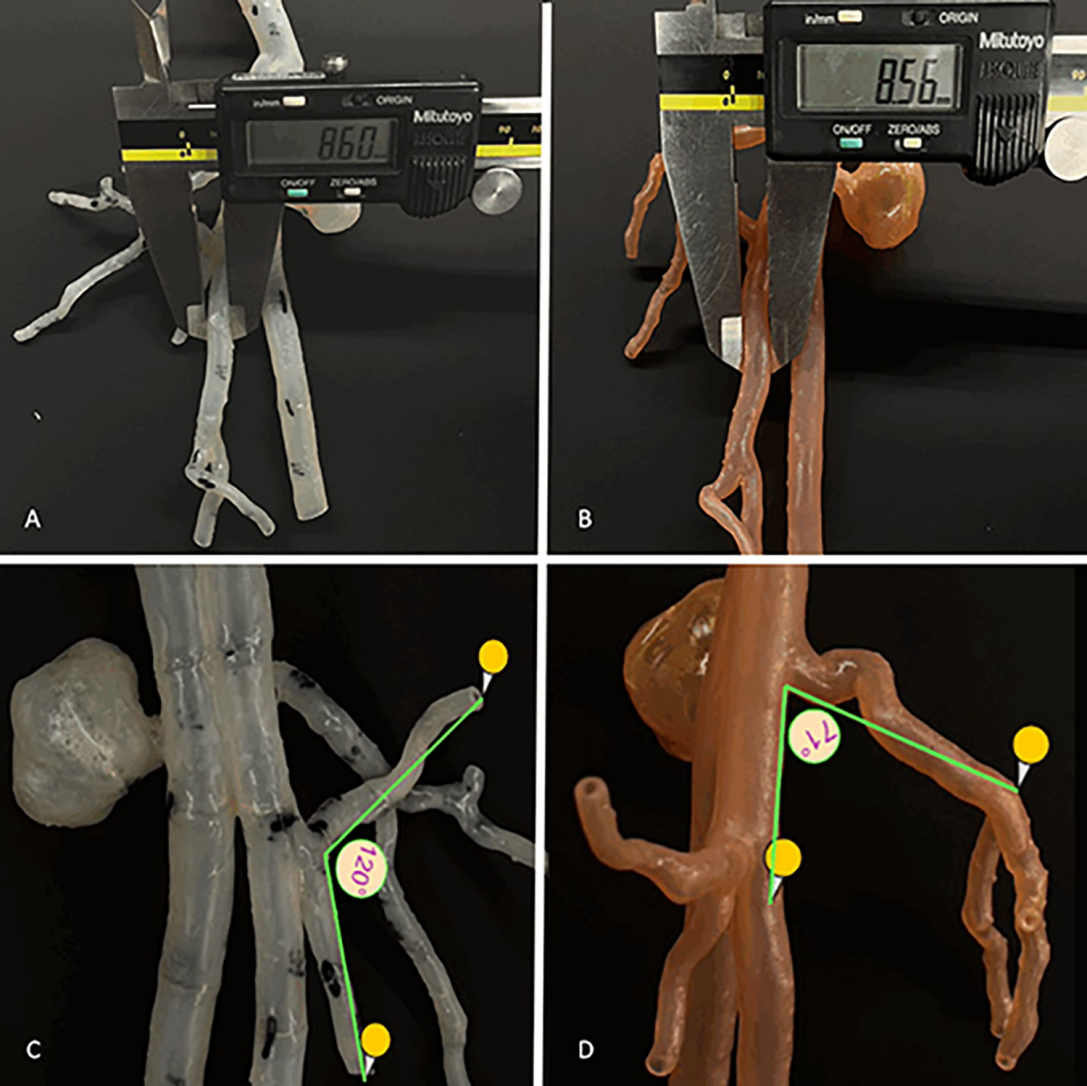
The measurement of the diameter at anatomical location 6 for **(A)** 3D model 1 and **(B)** 3D model 2. It also displays the measurement of the angle at anatomical location 8 for **(C)** 3D model 1 and anatomical location 3 for **(D)** 3D model 2.

In the current study, two 3D-printed models were created **(Fig 2)**, and a series of comparisons were conducted between the 3D segmented images from CT scans and these 3D-printed models. The objective was to assess the accuracy and feasibility of the 3D-printed models. For each 3D-printed model, a total of ten comparisons involving internal diameters **(Fig 3A)** and ten comparisons involving angles **(Fig 3B)** of the specific anatomical location **(Table 1)** were measured. The measurements were performed three times by two independent observers, and the mean value was used to reduce bias. The diameter and angle of the 3D segmented model were measured using tools in Materialise Mimics 22.0 software, while the 3D-printed models were evaluated using a digital millimeter caliper and digital protractor **(Fig 4).** Furthermore, we compared the absolute mean difference between the segmented model and the 3D-printed model using a T-test.

**Table 1:** Measurement of ten specific anatomical location in diameter(mm) for the 3D segmented model, printing model 1 and printing model 2.

Measurement Location for Diameter(mm)	Measurement Location for Angle(°)
1 = Common femoral artery	1 = Bifurcation between deep profunda femoral artery and superficial femoral artery
2 = Bifurcation site common femoral artery	2 = Superior angle between lateral femoral circumflex artery and deep profunda femoral artery
3 = Proximal segment of lateral femoral circumflex artery	3 = Inferior angle between lateral femoral circumflex artery and deep profunda femoral artery
4 = Middle segment of lateral femoral circumflex artery	4 = Superior angle between 1st perforating artery and deep profunda femoral artery
5 = Proximal segment of deep profunda femoral artery	5 = Inferior angle of birfucation between 1st perforating artery and deep profunda femoral artery
6 = Middle segment of deep profunda femoral artery	6 = Superior angle between middle femoral circumflex artery and deep profunda femoral artery
7 = Proximal superficial femoral artery	7 = Inferior angle between middle femoral circumflex artery and deep profunda femoral artery
8 = Middle superficial femoral artery	8 = Bifurcation of middle femoral circumflex artery
9 = Transverse largest diameter of aneurysm	9 = Superior angle between neck of aneurysm and the superficial femoral artery
10 = Longitudinal largest diameter of aneurysm	10 = Inferior angle between neck of aneurysm and the superficial femoral artery

### Questionnaires to assess the utility of the 3D-printed model for education

To validate the effectiveness and feasibility of our 3D-printed model as a simulator for endovascular training, we held a user trial during a day-long introduction to radiology residents and consultants. We enrolled nine residents and four consultants from this event on a voluntary basis. After 30 minutes for an introduction to the application of the 3D-printed pseudoaneurysm model, each participant was given 20–30 minutes to complete a simulated endovascular procedure on the 3D-printed model that connected to a pulse generator, including femoral angiography and introducing the microcatheter (2.7-Fr PROGREAT) and microwire(GLIDEWIRE^®^ GT) into the pseudoaneurysm sac for embolization with coils(Nylon fibered coils). Immediately after the simulation session, participants were asked to complete anonymous feedback questionnaire for evaluating their experience with the 3D-printed model. The questionnaire consisted of twelve questions with Likert-type scale responses.

Overall, our approach allowed for a comprehensive assessment of the accuracy and feasibility of the 3D-printed models.

## Result

### Difference in diameter and angle between 3D segmented images and 3D-printed models

The study measured the difference in diameter **(Table 2)** and angle **(Table 3)** between 3D segmented images from CT and two 3D-printed models at ten specific anatomical locations. The results showed the absolute mean difference in diameter between the 3D segmented images and 3D-printed model 1 was 0.18±0.15 mm, and for 3D-printed model 2, it was 0.22±0.14mm. There was no significant difference in the absolute mean diameter difference between the two sets of models (P = 0.572). Additionally, the absolute mean difference in angle between the 3D segmented images and 3D-printed model 1 was 0.99±0.65°, and for 3D-printed model 2, it was 1.00±0.91°. The absolute mean difference in angle between the two sets of models was also not significant (P = 0.962).

**Table 2:** Measurement of ten specific anatomical location in diameter(mm) for the 3D segmented model, printing model 1 and printing model 2.

Location	3D model (mm)	Printing Model 1(mm)	Printing Model 2(mm)
**1**	12.31	12.32	12.43
**2**	13.01	12.63	13.37
**3**	6.63	6.45	6.48
**4**	5.25	5.32	5.37
**5**	11.07	10.9	10.77
**6**	8.5	8.6	8.56
**7**	11.72	11.41	11.34
**8**	10.26	10.29	10.27
**9**	27.58	27.7	27.83
**10**	28.61	29.03	29.02

**Table 3:** Measurement of ten specific anatomical location in angle(°) for the 3D segmented model, printing model 1 and printing model 2.

Location	3D model (°)	Printing Model 1°)	Printing Model 2°)
**1**	14.05	14.46	14.12
**2**	90.31	89.86	91.32
**3**	72.76	71.01	70.51
**4**	118.13	119.38	118.54
**5**	53.55	52.19	52.1
**6**	88.26	89.55	88.6
**7**	16.91	16.07	18.5
**8**	123.7	121.68	123.81
**9**	88.08	88.1	87.83
**10**	56.85	57.32	54.3

### Bland-Altman analysis—Accuracy of 3D printing

**Fig 5:**
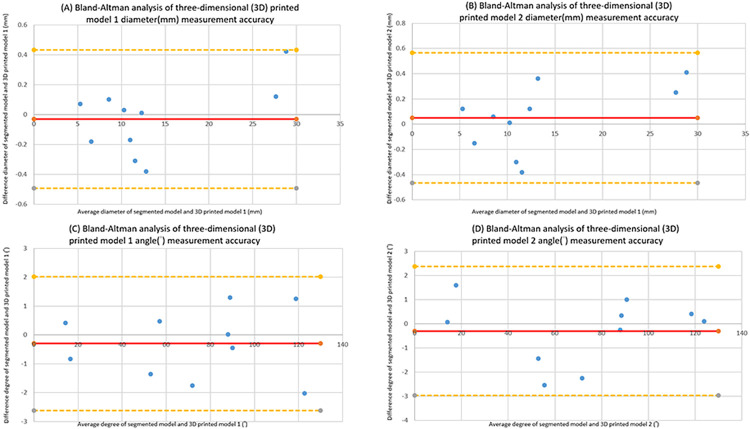
Bland-Altman analysis of three-dimensional (3D) printed model measurement accuracy. Graph A and B show the diameter measurements agreement between segmented model (.stl format data derived from CT data) and 3D printed model 1 **(A)** and 3D printed model 2 **(B)** at ten specific anatomical locations. Values are expressed in mm. Graph C and D show the angle measurements agreement between segmented model (STL format data derived from CT data) and 3D printed model 1 **(C)** and 3D printed model 2 **(D)** at ten specific anatomical locations. Values are expressed in degree(°). Central red line represents mean bias of difference. Yellow dotted lines represent upper and lower limits of agreement (LOA) ± 1.96 standard deviation.

The accuracy of 3D printing was further confirmed through Bland-Altman analysis **(Fig 5)**, which demonstrated a high correlation in dimension measurements between the 3D-printed models and segmented images. Specifically, there was a small mean bias and standard deviation in diameter (-0.03 ± 0.24 mm for model 1 and 0.05 ± 0.26mm for model 2) and angle (-0.30 ± 1.19° for model 1 and -0.30 ± 1.36° for model 2), indicating that the 3D-printed models accurately replicated the anatomical details of the segmented images. Specifically, the mean bias for the two-set data was very small for both diameter and angle measurements respectively, indicating a high degree of accuracy in replicating the actual size of the patient anatomy. The standard deviation values were also relatively small, indicating a low degree of variability in the measurements. Overall, these results suggest that 3D printing is an accurate method for creating 3D anatomical models that closely replicate patient anatomy from CT images.

### Fidelity and application of the 3D-printing model

**Fig 6:**
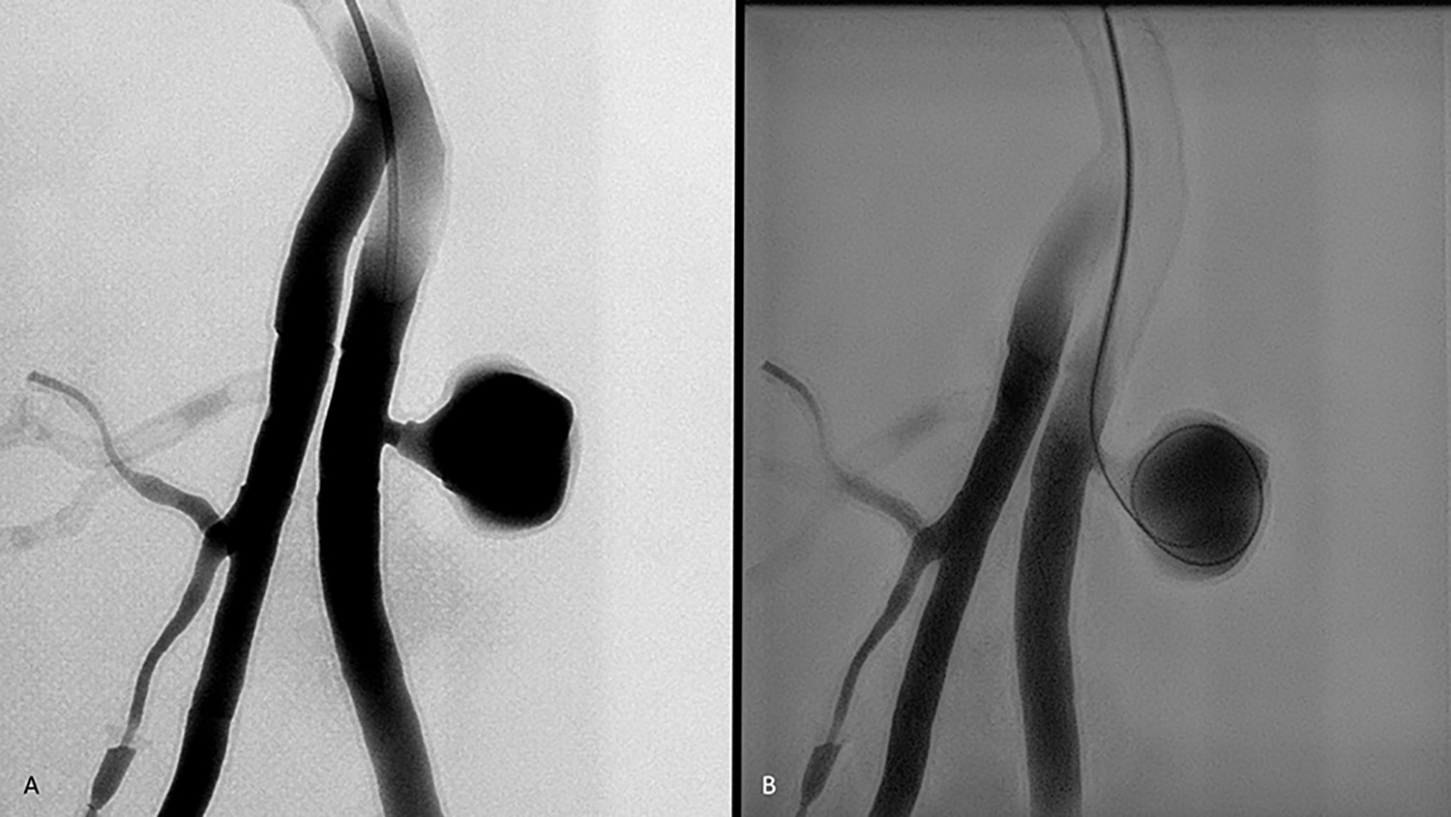
**(A)** Digital subtraction angiography (DSA) performed by injection of contrast into the 3D-printed model, revealing a pseudoaneurysm which resembles the real case. **(B)** Coil embolization of the pseudoaneurysm sac performed under fluoroscopic guidance by Philips Azurion7 in the angiography room.

Using the Philips Azurion7 image-guided therapy platform in the angiography room, the accuracy of a 3D-printed pelvic vascular model was further tested through the simulation of a superficial femoral artery pseudoaneurysm coiling procedure. The model was accessed via a 6-Fr sheath, and a catheter(5-Fr C1 catheter) was inserted through the sheath coaxially with a guidewire. Digital subtraction angiography (DSA) was then performed by injecting contrast (Iohexol, GE; rate 3ml/s and total volume 15ml) into the model, revealing a pseudoaneurysm that closely resembled a real-life case **(Fig 6A)**. Subsequently, coil embolization with twelve nylon fibered coils of the pseudoaneurysm sac was performed under fluoroscopic image guidance **(Fig 6B) through microcatheter**. The simulation successfully demonstrated that the pelvic vascular model was able to replicate the tactile sensation and procedural feel of a real-life intervention, as confirmed by the images captured during the procedure.

In total, 13 subjects completed a post-trial questionnaire rating their agreement with twelve statements regarding their training experience on a standard 5-point Likert scale following the course using this 3D-printed model.

An overview of the responses to the questions according to realism, acceptability, and feasibility for the procedure training with this 3D-printed model is given **(Table 4).**

**Table 4:** An overview of the responses to the questionnaire.

Question	n	Strongly disagree	Disagree	Neither agree nor disagree	Agree	Strongly agree	Mean (SD)
**I enjoyed this training method**	13	0	0	0	5	8	4.615(0.506)
**The 3D-printed model is user-friendly?**	13	0	0	3	4	6	4.230(0.832)
**I feel more confident about my skills after this training**	13	0	0	0	6	7	4.538(0.519)
**This 3D-printed model is a good addition to my current training**	13	0	0	0	3	10	4.769(0.439)
**The simulation of the procedure using 3D-printed model was realistic**	13	0	0	4	6	3	3.923(0.760)
**The haptic feedback from the 3D-printed model was realistic**	13	0	0	2	7	4	4.153(0.689)
**I could demonstrate my skills effectively**	13	0	0	4	3	6	4.153(0.899)
**I have better understanding of this procedure after this training**	13	0	0	0	4	9	4.692(0.480)
**This 3D-printed model could enhance my understanding of technique of vascular access and catheter placement**	13	0	0	0	4	9	4.692(0.480)
**I could manipulate guide wire and catheter effectively on this 3D-printed model**	13	0	0	2	6	5	4.231(0.725)
**I could catheterization the target vessel or the pseudoaneurysm sac effectively on this 3D-printed model**	13	0	0	3	6	4	4.077(0.766)
**I would like to use 3D printed model again for endovascular training**	13	0	0	0	0	13	5(0)

## Discussion

The utilization of 3D-printed models has gained recognition as a valuable educational tool for learning anatomy and pathology. Moreover, they have proven to be of great value in assisting surgical planning and simulation of various procedures, thereby enhancing surgical care and patient management. The precision and accuracy of 3D-printed models, which are created using patient anatomy or segmented images, plays a pivotal role in ensuring the efficacy of surgical planning, education and training, effective patient communication, and maintaining patient safety across a spectrum of medical applications [13–27]. The result of our study further affirms the reliability of 3D-printed models created based on CT images.

Producing a 3D-printed model for medical training involves a series of crucial steps and procedures. The initial phase entails selecting a case featuring a specific disease or pathology. [17] Image acquisition using rotational DSA, MRI, or CT with contrast is then necessary to generate high-quality 3D images in specialized software [18]. Specific imaging guidelines can help optimize the accuracy of converting 2D images to a 3D object. After acquiring the imaging data set, it is transferred into a 3D printing software for segmentation, processing, and creation of an STL file that can be printed.

In our study, we used Materialise Mimics, an FDA-approved software, for segmenting the pelvic endovascular model. To ensure accurate segmentation, only relevant series, or preferred slices of images from each acquisition should be imported into the 3D printing software due to data storage limitations and the risk of misinterpretation by the software. In our case, artery phase of the pelvic CT to ensure the pseudoaneurysm sac was well depicted and specified images were obtained from the level of abdominal aorta near the aortic bifurcation to the femoral segment containing the superficial femoral artery pseudoaneurysm. Finally, before printing, a CAD software is used to create an STL file.

The exported STL file from segmentation software may contain errors and is typically not optimal for printing, thus requiring repair and refinement in CAD software. In CAD software, post-processing steps such as fixing, wrapping, and smoothing, as well as co-registration of multiple model parts can be performed to ensure that the CAD model is in a printable state. There are several CAD software options available for mesh editing, including Meshmixer (Autodesk, Inc., San Rafael, CA), Meshlab (available at https://www.meshlab.net/), Thinkercad (AutoDesk® Inc., USA), and FreeCAD (open source, LGPL license) [19]. While segmentation and STL-refinement CAD are two distinct categories of software, software suites such as Mimics Innovation Suite (Materialise, Leuven, Belgium) and Mimics inPrint (Materialise, Leuven, Belgium) offer solutions to both functions. In our study, we utilized the Mimics Innovation Suite, which includes Materialise Mimics and Materialise 3-Matic for this 3D printing simulation model [19].

According to the American Society for Testing and Materials (ASTM), there are seven types of 3D printing methods, including vat polymerization, material jetting, binder jetting, material extrusion, powder bed fusion, sheet lamination, and directed energy deposition [20, 21]. In our study, vat polymerization was used for printing due to its ability to print hollow vessel lumens without solid support material by orienting them straight to avoid internal support inside the vessels that are difficult to remove, especially in small, long or tortuous vessels. Additionally, vat polymerization is an accurate printing method that enables a 3D model to be printed with small vessel structures.

The accuracy of 3D printing in replicating patient anatomy has been investigated in studies dating back to 1994, such as the one conducted by Barker et al. [22] In their study, a dry skull was imaged using CT, and the resulting images were used to create a segmented bone model that was then 3D-printed using state-of-the-art software and hardware at that time. The study found that the average difference of 11 distances between anatomic landmarks measured on the cadaveric bone and those measured on the 3D-printed model using a caliper was 1.8 mm, with a range of 0.10–4.62 mm (0.6%-3.7%). This demonstrated the relatively small margin of error in the accuracy of the 3D printing process in replicating patient anatomy.

Several studies in the literature [23, 24], in addition to the work by Barker et al, have investigated the accuracy of 3D-printed models by comparing them with the raw data of segmented models using various 3D printing technologies. These studies have assessed the ability of 3D printing to replicate patient anatomy for a wide range of anatomical structures, including the skull, mandible, vertebral bodies, pelvis, hearts, and arteries [25–33]. By evaluating the accuracy of 3D printing in replicating patient anatomy, these studies [25–33] contribute to our understanding of the reliability and precision of 3D-printed models in medical applications.

The accuracy of 3D-printed models in replicating anatomical details is crucial for their reliability and usefulness in various applications. Recent research [34–42] has shown that there is a minimal difference between 3D-printed models and original source imaging data, regardless of the imaging modality used, such as CT, echocardiography, MRI, or rotational angiography. Comparisons between 3D-printed models and original source images, based on current literature, have revealed a mean difference in dimensional measurements of less than 0.5 mm, indicating a high level of accuracy in the 3D models.

Furthermore, our study goes beyond the typical measurement of length to evaluate the accuracy of 3D printing models. We assessed the orientation and degree of specific anatomy structures by measuring the angle at ten specific anatomical locations, which enabled us to determine the orientation of the vascular branches. Our results show that the 3D-printed models not only accurately replicated the length of the segmented model but also the orientation of the specific anatomical structure. We observed a mean difference in degree measurements of -0.30 ± 1.19° for model 1 and -0.30 ± 1.36° for model 2, indicating good correlation between the 3D-printed models and the segmented model. As a result, excellent correlations have been observed between different observers and measurement approaches, highlighting the reliability and consistency of 3D-printed models in replicating patient anatomy across different imaging modalities.

The challenge of using this method in printing this model is the process in removing the supporting material without breaking the model. Vat polymerization is a printing method with three components including a high intensity light source, vat that holds a photo-curable liquid resin and controlling system which directs the light source to illuminate a specific area of resin. After a layer of specific area of resin is exposed to light and cured, the model is lowered or raised one layer of thickness to cover the previous printed layer. Each layer thickness is thus printed sequentially until the final layer is completed. After the printing is finished, post-processing to refine the model is necessary [43]. Post-processing of our printed model included removing the undesired support structures and the model is rinsed in a wash solution (isopropyl alcohol) to get rid of the liquid layer of resin. Subsequent steps including curing the model with ultraviolet light to enhance its mechanical properties.

Our study has certain limitations that need to be addressed. Firstly, unlike traditional animal models, our endovascular model does not allow for coil embolization with blood clotting as there is no integrated *clot* promoting activity in our model. Secondly, pulsatile simulator used in our study was unable to simulate the native pressure and pulsatility of flowing blood naturally. Furthermore, while our study focuses on creating an educational model to assess its accuracy and feasibility for education, there is a lack of designs and evaluation to determine the educational impact of this training model although questionnaires was performed among the participants. Future studies can address this by designing a course or workshop to gather more specific feedback from participants. Additionally, although our superficial femoral artery pseudoaneurysm model is suitable for endovascular training, it is not performed for stenting in our study [44] and currently non-applicable for diagnostic training [45] with ultrasound and other treatment methods such as ultrasound-guided compression or duplex-guided thrombin injection. Therefore, it is necessary to integrate this vascular model into an ultrasound-guided training model to encompass training in other treatment methods using ultrasound. Furthermore, the 3D model might lack flexibility and rigidity when compared to the blood vessel which could restrict the change of their angles or curves while introducing catheters. Moreover, it’s crucial to acknowledge that the accuracy of measurements is inherently tied to the precision and reliability of the instruments used. Therefore, there are potential limitations or inaccuracies associated with these measurement instruments. It is essential to acknowledge the preliminary nature of our work, as our investigation is based on the fabrication and measurement of accuracy using only two 3D-printed models. To thoroughly assess the reliability of the 3D printing process, future studies should aim to extend the sample size by printing more than two models. Additionally, the scope of accuracy testing should encompass a diverse range of designs and materials to provide a comprehensive understanding of the technique’s performance across various scenarios. The current study, limited to two models, serves as an initial exploration, and the broader generalization of results requires further validation through expanded experimental designs. Another limitation to consider is that our 3D-printed model is single use for endovascular embolization treatment training, which means that a new model would need to be printed for each training session. This can be costly, especially if multiple training sessions are required. Therefore, cost-effectiveness and sustainability of using 3D-printed models for endovascular training should be further evaluated.

## Conclusions

In conclusion, the 3D-printed superficial femoral artery pseudoaneurysm model presented in this article not only exhibits a commendable level of accuracy but also demonstrates practicality and feasibility for endovascular training when compared to real-life cases. Notably, this study marks a significant milestone as the first instance of developing a superficial femoral artery pseudoaneurysm 3D-printed model with such precision, encompassing accurate measurements of length, orientation, and specific anatomical structures. The manufacturing process can serve as a valuable template for the creation of other endovascular 3D models for simulation training purposes. However, it is important to refine the limitations mentioned above and quantitatively evaluate the educational effect of this model in future studies. Despite these constraints, our findings assert that 3D printing technology, particularly the creation of models from CT images, is not only reliable and accurate but also represents a feasible and effective educational method in medical training. Our results affirm the feasibility and effectiveness of utilizing 3D-printed models for endovascular training, paving the way for further advancements in medical education methodologies. The development and integration of 3D printing technology in medical education has the potential to improve surgical planning, patient communication, and ultimately, patient outcomes.

## References

[1] BAIRD RJ, DORAN ML. THE FALSE ANEURYSM. Can Med Assoc J. 1964 Aug 8;91(6):281–4. 14180533 PMC1927240

[2] Dwivedi K, Regi JM, Cleveland TJ, et al. Long-Term Evaluation of Percutaneous Groin Access for EVAR. Cardiovasc Intervent Radiol 2019; 42:28. doi: 10.1007/s00270-018-2072-3 30288590 PMC6267668

[3] Stone PA, Campbell JE, AbuRahma AF. Femoral pseudoaneurysms after percutaneous access. J Vasc Surg. 2014 Nov;60(5):1359–1366. doi: 10.1016/j.jvs.2014.07.035 25175631

[4] Moonen HPFX, Koning OHJ, van den Haak RF, et al. Short-term outcome and mid-term access site complications of the percutaneous approach to endovascular abdominal aortic aneurysm repair (PEVAR) after introduction in a vascular teaching hospital. Cardiovasc Interv Ther 2019; 34:226. doi: 10.1007/s12928-018-0547-4 30259385

[5] Standard for diagnostic arteriography in adults. Standards of Practice Committee of the Society of Cardiovascular and Interventional Radiology.*J Vasc Interv Radiol*. 1993; 4: 385–395 8513213

[6] Katzenschlager R, Ugurluoglu A, Ahmadi A, Hülsmann M, Koppensteiner R, Larch E, et al. Incidence of pseudoaneurysm after diagnostic and therapeutic angiography. Radiology. 1995 May;195(2):463–6. doi: 10.1148/radiology.195.2.7724767 7724767

[7] Moll R, Habscheid W, Landwehr P. Häufigkeit des Aneurysma spurium der Arteria femoralis nach Herzkatheteruntersuchung und PTA [The frequency of false aneurysms of the femoral artery following heart catheterization and PTA (percutaneous transluminal angioplasty)]. Rofo. 1991 Jan;154(1):23–7. German. 1846687 10.1055/s-2008-1033079

[8] Hirano Y, Ikuta S, Uehara H, Nakamura H, Taniguchi M, Kimura A, et al. [Diagnosis of vascular complications at the puncture site after cardiac catheterization]. J Cardiol. 2004 Jun;43(6):259–65. Japanese. 15242075

[9] Noori VJ, Eldrup-Jørgensen J. A systematic review of vascular closure devices for femoral artery puncture sites. J Vasc Surg 2018; 68:887. doi: 10.1016/j.jvs.2018.05.019 30146036

[10] Lamelas J, Williams RF, Mawad M, LaPietra A. Complications Associated With Femoral Cannulation During Minimally Invasive Cardiac Surgery. Ann Thorac Surg 2017; 103:1927. doi: 10.1016/j.athoracsur.2016.09.098 28017338

[11] Hajibandeh S, Hajibandeh S, Antoniou SA, et al. Percutaneous access for endovascular aortic aneurysm repair: A systematic review and meta-analysis. Vascular 2016; 24:638. doi: 10.1177/1708538116639201 27000385

[12] Vierhout BP, Pol RA, El Moumni M, Zeebregts CJ. Editor’s Choice—Arteriotomy Closure Devices in EVAR, TEVAR, and TAVR: A Systematic Review and Meta-analysis of Randomised Clinical Trials and Cohort Studies. Eur J Vasc Endovasc Surg 2017; 54:104. doi: 10.1016/j.ejvs.2017.03.015 28438400

[13] Coles-Black J, Bolton D, Chuen J. Accessing 3D Printed Vascular Phantoms for Procedural Simulation. Front Surg. 2021 Jan 27;7:626212. doi: 10.3389/fsurg.2020.626212 33585550 PMC7873568

[14] Mafeld S, Nesbitt C, McCaslin J, Bagnall A, Davey P, Bose P, et al. Three-dimensional (3D) printed endovascular simulation models: a feasibility study. Ann Transl Med. 2017 Feb;5(3):42. doi: 10.21037/atm.2017.01.16 28251121 PMC5326638

[15] Ye Z, Dun A, Jiang H, Nie C, Zhao S, Wang T, et al. The role of 3D printed models in the teaching of human anatomy: a systematic review and meta-analysis. BMC Med Educ. 2020 Sep 29;20(1):335. doi: 10.1186/s12909-020-02242-x 32993608 PMC7523371

[16] Goudie C, Kinnin J, Bartellas M, et al. The Use of 3D Printed Vasculature for Simulation-Based Medical Education within Interventional Radiology. Cureus 2019;11(4):e4381. doi: 10.7759/cureus.4381 31218145 PMC6553672

[17] De Backer P, Allaeys C, Debbaut C, et al. Point-of-Care 3D Printing: A Low-Cost Approach to Teaching Carotid Artery Stenting. 3D Print Med 2021;7(1. doi: 10.1186/s41205-021-00119-3 34476605 PMC8414696

[18] Haleem A, Javaid M, Suman R, et al. 3D Printing Applications for Radiology: An Overview. Indian J Radiol Imaging 2021;31(1):10–17. doi: 10.1055/s-0041-1729129 34316106 PMC8299499

[19] Rybicki FJ and Grant GT, (eds). 3D Printing in Medicine: A Practical Guide for Medical Professionals. 1st ed. Springer International Publishing: Basel, Switzerland; 2017.

[20] Alexander AE, Wake N, Chepelev L, Brantner P, Ryan J, Wang KC. A guideline for 3D printing terminology in biomedical research utilizing ISO/ASTM standards. 3D Print Med 2021;7(1):8. doi: 10.1186/s41205-021-00098-5 33751279 PMC7986506

[21] Huang Y, Leu MC. Frontiers of Additive Manufacturing Research and Education. http://nsfam.mae.ufl.edu/2013NSFAMWorkshopReport.pdf. Published March 2014. Accessed December 7, 2021.

[22] Additive Manufacturing Technology Standards. ASTM International. https://www.astm.org/Standards/additivemanufacturing-technology-standards.html. Accessed July 7, 2021.

[23] Bastawrous S, Wu L, Liacouras PC, Levin DB, Ahmed MT, Strzelecki B, et al. Establishing 3D Printing at the Point of Care: Basic Principles and Tools for Success. Radiographics. 2022 Mar-Apr;42(2):451–468. doi: 10.1148/rg.210113 35119967

[24] Barker TM, Earwaker WJ, Lisle DA. Accuracy of stereolithographic models of human anatomy. Australas Radiol. 1994 May;38(2):106–11. doi: 10.1111/j.1440-1673.1994.tb00146.x 8024501

[25] George E, Liacouras P, Rybicki FJ, Mitsouras D. Measuring and Establishing the Accuracy and Reproducibility of 3D Printed Medical Models. Radiographics. 2017 Sep-Oct;37(5):1424–1450. doi: 10.1148/rg.2017160165 28800287 PMC5621728

[26] Sun Z. Patient-Specific 3D-Printed Models in Pediatric Congenital Heart Disease. Children (Basel). 2023 Feb 7;10(2):319. doi: 10.3390/children10020319 36832448 PMC9955978

[27] Chang PS, Parker TH, Patrick CW Jr, Miller MJ. The accuracy of stereolithography in planning craniofacial bone replacement. J Craniofac Surg 2003;14(2):164–170.’ doi: 10.1097/00001665-200303000-00006 12621285

[28] Taft RM, Kondor S, Grant GT. Accuracy of rapid prototype models for head and neck reconstruction. J Prosthet Dent 2011;106(6):399–408. doi: 10.1016/S0022-3913(11)60154-6 22133397

[29] Petropolis C, Kozan D, Sigurdson L. Accuracy of medical models made by consumer-grade fused deposition modelling printers. Plast Surg (Oakv) 2015;23(2):91–94. doi: 10.4172/plastic-surgery.1000912 26090349 PMC4459415

[30] Ibrahim D, Broilo TL, Heitz C, et al. Dimensional error of selective laser sintering, three-dimensional printing and PolyJet models in the reproduction of mandibular anatomy. J Craniomaxillofac Surg 2009;37(3):167–173. doi: 10.1016/j.jcms.2008.10.008 19056288

[31] Choi JY, Choi JH, Kim NK, et al. Analysis of errors in medical rapid prototyping models. Int J Oral Maxillofac Surg 2002;31(1):23–32. doi: 10.1054/ijom.2000.0135 11936396

[32] Silva DN, Gerhardt de Oliveira M, Meurer E, Meurer MI, Lopes da Silva JV, Santa-Bárbara A. Dimensional error in selective laser sintering and 3D-printing of models for craniomaxillary anatomy reconstruction. J Craniomaxillofac Surg 2008;36(8):443–449. doi: 10.1016/j.jcms.2008.04.003 18579391

[33] van Eijnatten M, Rijkhorst EJ, Hofman M, Forouzanfar T, Wolff J. The accuracy of ultrashort echo time MRI sequences for medical additive manufacturing. Dentomaxillofac Radiol 2016;45(5):20150424. doi: 10.1259/dmfr.20150424 26943179 PMC5084702

[34] Ogden KM, Aslan C, Ordway N, Diallo D, Tillapaugh-Fay G, Soman P. Factors affecting dimensional accuracy of 3-D printed anatomical structures derived from CT data. J Digit Imaging 2015;28(6):654–663. doi: 10.1007/s10278-015-9803-7 25982877 PMC4636725

[35] Wu XB, Wang JQ, Zhao CP, et al. Printed three-dimensional anatomic templates for virtual preoperative planning before reconstruction of old pelvic injuries: initial results. Chin Med J (Engl) 2015;128(4):477–482. doi: 10.4103/0366-6999.151088 25673449 PMC4836250

[36] Lee S.; Squelch A.; Sun Z. Quantitative Assessment of 3D Printed Model Accuracy in Delineating Congenital Heart Disease.Biomolecules 2021, 11, 270. doi: 10.3390/biom11020270 33673159 PMC7917618

[37] Valverde I.; Gomez-Ciriza G.; Hussain T.; Suarez-Mejias C.; Velasco-Forte M.N.; Byrne N.; et al. Three-dimensional printed models for surgical planning of complex congenital heartdefects: An international multicentre study. Eur. J. Cardio-Thoracic Surg. 2017, 52, 1139–1148. 10.1093/ejcts/ezx208 28977423

[38] Olejník P.; Nosal M.; Havran T.; Furdova A.; Cizmar M.; Slabej M.; et al. Utilisation of three-dimensional printed heart models for operative planning of complex congenital heart defects. Kardiol. Pol. 2017, 75, 495–501. doi: 10.5603/KP.a2017.0033 28281732

[39] Olivieri L.J.; Krieger A.; Loke Y.-H.; Nath D.S.; Kim P.C.; Sable C.A. Three-Dimensional Printing of Intracardiac Defects from Three-Dimensional Echocardiographic Images: Feasibility and Relative Accuracy. J. Am. Soc. Echocardiogr. 2015, 28, 392–397. doi: 10.1016/j.echo.2014.12.016 25660668

[40] Lau I.W.W.; Liu D.; Xu L.; Fan Z.; Sun Z. Clinical value of patient-specific three-dimensional printing of congenital heart disease: Quantitative and qualitative assessments. PLoS ONE 2018, 13, e0194333. doi: 10.1371/journal.pone.0194333 29561912 PMC5862481

[41] Mowers K.L.; Fullerton J.B.; Hicks D.; Singh G.K.; Johnson M.C.; Anwar S. 3D echocardiography provides highly accurate 3Dprinted models in congenital heart disease. Pediatr. Cardiol. 2021, 42, 131–141. doi: 10.1007/s00246-020-02462-4 33083888

[42] Parimi M.; Buelter J.; Thanugundla V.; Condoor S.; Parkar N.; Danon S.;et al. Feasibility and Validity of Printing 3D HeartModels from Rotational Angiography. Pediatr. Cardiol. 2018, 39, 653–658. 10.1007/s00246-017-1799-y 29305642

[43] Pagac M, Hajnys J, Ma Q-P, et al. A Review of Vat Photopolymerization Technology: Materials, Applications, Challenges, and Future Trends of 3D Printing. Polymers (Basel) 2021;13(4):598. doi: 10.3390/polym13040598 33671195 PMC7922356

[44] Tsai Chien-Chung, Chiu Kuan-Chi, et al. Endovascular Treatment of Peripheral Pseudoaneurysms Using a Wallgraft Stent Prothesis. Chinese Journal of Radiology(Taiwan) 2006; 31: 297–302

[45] Gueng Mein-Kai, Chou Ting-Ywan, Wu Chin-Jiunn. Evaluation of lower extremity peripheral arterial disease with color doppler ultrasound: comparison with angiography. Chinese Journal of Radiology(Taiwan) 1995; 20(2): 129–137

